# Deciphering the Complexities of Sodium Voltage-Gated Channel Alpha Subunit 1 (SCN1A) Mutation: A Case of Intractable Epilepsy in a Five-and-a-Half-Month-Old Male

**DOI:** 10.7759/cureus.64171

**Published:** 2024-07-09

**Authors:** Indrayani Jadhav, Keta Vagha, Ashish Varma, Jayant D Vagha, Yash V Lath, Jaywant Jadhav

**Affiliations:** 1 Pediatrics, Jawaharlal Nehru Medical College, Datta Meghe Institute of Higher Education and Research, Wardha, IND

**Keywords:** epileptic encephalopathy, prognosis, personalized medicine, genetic testing, intractable epilepsy, nav1.1 protein, scn1a mutation

## Abstract

If the sodium voltage-gated channel alpha subunit 1 (*SCN1A*) gene, which encodes Nav1.1 protein, undergoes pathological mutation, it results in a wide range of epileptic syndrome, including febrile seizure, genetic epilepsy with febrile seizure plus (GEFS+), and developmental and epileptic encephalopathy (DEE), including Dravet syndrome. We present the case of a five-and-a-half-month-old boy with *SCN1A* gene-related epileptic seizures, starting as focal seizures and progressing to generalized tonic-clonic seizures. Despite treating the seizures with multiple antiepileptic drugs, including phenytoin, sodium valproate, levetiracetam, perampanel, and clobazam, it was very difficult to control the seizures, and genetic testing was suggested. The *SCN1A* mutation leads to either loss of function, including GEFS+ and Dravet syndrome, or gain of function, including familial hemiplegic migraine type 3. The case emphasizes the importance of genetic testing in refractory epilepsy management to provide medical strategies for the diagnosis. It focuses on the difficulties faced in diagnostic and treatment strategies for the management of *SCN1A*-related epilepsy. It emphasizes the importance of monitoring and personalized treatment strategies to reduce the incidence of refractory epilepsy.

## Introduction

Epilepsies are a compilation of a wide variety of neurological conditions characterized by repeated, spontaneous seizures due to abnormal electrical activity in the brain. Many epilepsies are secondary to injury, or any other disease, but about 35-40% are idiopathic, which are believed to be a result of genetic abnormalities with a gene mutation. Most genes encode voltage-gated sodium, potassium, calcium, and chloride channels [1].

Voltage-gated sodium channels are responsible for initiating action potential in brain neurons. Sodium voltage-gated channel alpha subunit 1 (*SCN1A*) gene encoding by Nav1.1 protein is most commonly associated with mutation in the voltage-gated sodium channel [2]. Mutation in the *SCN1A* gene is linked to a broad spectrum of neurological disorders. These mutations can lead to conditions such as simple febrile seizures, genetic epilepsy with febrile seizures plus (GEFS+), and more severe developmental and epileptic encephalopathies (DEE), including Dravet syndrome [3].

The incidence of *SCN1A* gene mutations in the United States has been estimated at 1 in 40,000 [4]. Studies suggest that 70-80% of patients suffering from Dravet syndrome, a severe form of epilepsy, have a mutation in the *SCN1A* gene [5]. More specifically, the prevalence of the disease ranges from 1 in 15,700 to 1 in 40,000 live births, affecting both male and female children equally. GEFS+ is also associated with a mutation in the *SCN1A* gene, but the prevalence is not well-defined due to broad-spectrum disorder [6].

Nav1.1 protein encoded by the *SCN1A* gene, expressed predominantly in GABAergic neurons, is responsible for the initiating and propagating action potential. It prevents the unnecessary hyperexcitation of neurons. With mutation in the *SCN1A* gene, this inhibitory action is lost, increasing the risk of seizure activity [7].

This case report emphasizes the diagnostic process and treatment strategies for a five-and-a-half-month-old child presenting with intractable epilepsy. This report also stresses the importance of genetic testing in children for accurate medical treatment to avoid any harmful diagnostic procedure and management. Additionally, it provides valuable information on the severity and prognosis of the disease [8].

## Case presentation

A five-and-a-half-month-old male, born to a 40-year-old primigravida via in vitro fertilization due to primary infertility in a non-consanguineous marriage, was delivered via lower-segment cesarean section weighing 2.7 kg. The infant cried immediately after birth and demonstrated normal development. There was no history of convulsions in the family. He initially presented with focal tonic-clonic convulsions in his right leg, which went unnoticed by the parents for the first two days but increased in frequency after that. Subsequently, he developed a fever and experienced generalized tonic-clonic seizure (GTCS) lasting for 15 minutes. Following hospitalization, he received intravenous levetiracetam and dextrose normal saline. The child was discharged with a prescription for levetiracetam (Levera) upon stabilization.

Three months later, the child had complaints of cough and cold during which the child had a similar convulsive episode lasting three minutes, controlled with midazolam nasal spray. Levetiracetam dosage was increased. Developmentally, he could sit with support, utter monosyllables, exhibit a social smile, and show no stranger anxiety. On examination, his vitals were normal, he responded to light, and his gross hearing was intact. However, he displayed microcephaly, mild hypertonia, grade 3+ deep tendon reflexes, and normal plantar reflexes. Over the next 10 months, he experienced multiple similar convulsive episodes, ranging from focal to multifocal to GTCS, often triggered by febrile illnesses. Various anticonvulsants were introduced and titrated with each episode, including phenytoin, valproate, perampanel, and clobazam.

**Figure 1:**
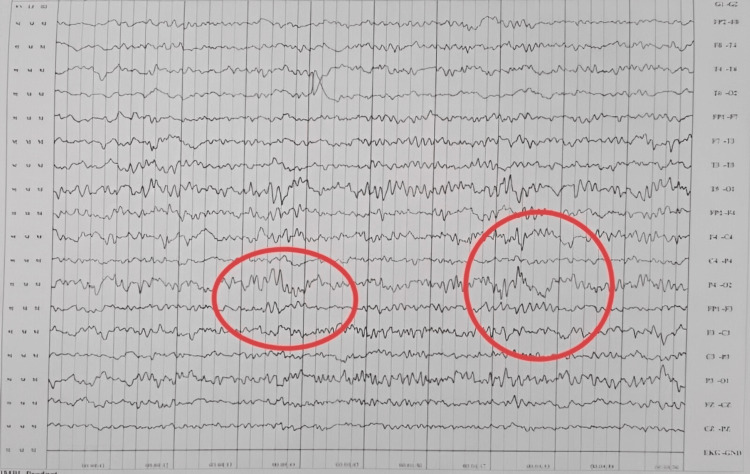
Electroencephalogram showing an abnormality in the form of bilateral fronto-central ictogenic discharge (red ring).

**Figure 2:**
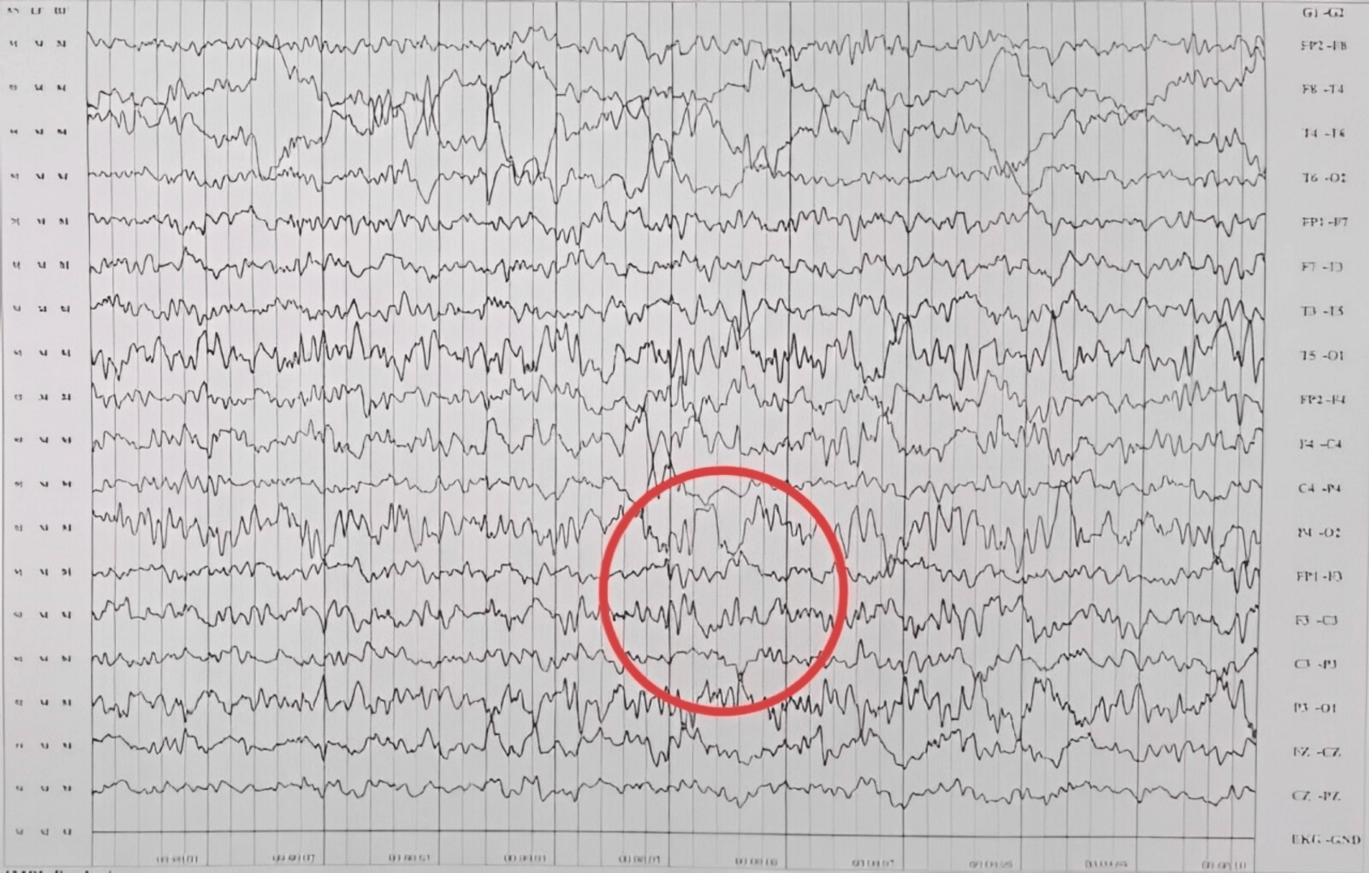
Electroencephalogram showing bilateral fronto-central ictogenic discharge (red ring).

There was a noticeable delay in the achievement of developmental milestones. Before the onset of convulsions, he achieved sitting with support at five months, sitting without support at 10 months, and crawling at 15 months. His speech development was limited to bisyllables. A repeat MRI at 15 months was normal, while electroencephalography (EEG) showed bilateral fronto-central ictogenic discharges (Figures 1, 2).

A tandem mass spectrometry to assess inborn errors of metabolism yielded normal results. Due to incomplete seizure control at six months of age, whole-exome sequencing revealed a heterozygous deletion in exon 8 of the *SCN1A* gene (ENST00000674923.1), identified as c.606del (p.Tyr202Ter), associated with developmental and epileptic encephalopathy-6B (OMIM#619317), Dravet syndrome (OMIM#619317), and generalized epilepsy with febrile seizures plus, type 2 (OMIM#619317)/familial febrile seizures-3A (OMIM#619317).

The parents were informed about the poor prognosis and advised to mitigate provoking factors. Subsequent weekly follow-ups showed decreasing seizure frequency but increasing hypertonia in all limbs, prompting regular physiotherapy and occupational therapy to improve cognition. At 18 months, he could creep, stand with support, play with toys, and speak single words with meaning.

## Discussion

The *SCN1A* gene, located on chromosome 2q24.3, holds pivotal significance in the pathogenesis of epilepsy. This gene encodes the alpha 1 subunit of the voltage-gated sodium channel Nav1.1, a 2,000 amino acid protein [9].

Globally, *SCN1A* mutations have a significant effect that results in epileptic disorders. The disease has variable prevalence and can appear in various forms of epilepsy, such as benign febrile seizures and severe DEE [3]. DEE has further been divided into clinical subtypes such as myoclonic atonic epilepsy, Dravet syndrome, epilepsy of infancy with migrating focal seizures, and early-onset *SCN1A* DEE [5].

The *SCN1A* gene is involved in producing Nav1.1 protein, which is expressed by GABAergic neurons releasing gamma-aminobutyric acid. This inhibitory neurotransmitter prevents neuronal hyperexcitability and neuronal overactivity, thereby playing a role in the body [10]. This protein causes sodium ions to rush into the neuron, initiating and propagating the action potential. Any defect in this protein’s actions leads to ineffective neuronal inhibition which then presents with hyperexcitability [2].

Any mutation in the *SCN1A* gene renders it incapable of coding for the production of Nav1.1 protein, which is associated with the loss of function (LOF) of the affected neurons. These neurons lack the potential to inhibit the neuronal network as there is reduced excitation of the GABAergic neurons. This phenomenon predisposes the individual to seizures [9]. Seizures of GEFS+ are well managed with normal cognition. However, in Dravet syndrome, with LOF, severe drug-resistant epilepsy can occur with intellectual disability [11].

Pathophysiologically, the disease presents with a change in functional properties, with either gain of function (GOF) or LOF of the affected neurons. LOF mutations, which cause Dravet syndrome and GEFS+, lead to hyperexcitability of neurons causing recurrent seizures [12]. Dravet syndrome is characterized by early-onset febrile seizures evolving into intractable epilepsy and cognitive impairment. At the same time, GOF variants manifest as familial hemiplegic migraine type 3, which presents as migraine headaches with aura [13].

In Dravet syndrome, seizures commence at an age below one year. The initial febrile seizures are followed by afebrile-resistant seizures accompanied by developmental delay and require the administration of multiple antiepileptic medications. GEFS+ usually presents as febrile seizures followed by febrile seizure plus (FS+). FS+ refers to children with febrile seizures who are not in classic febrile seizure limits (six months to six years) or when presented with afebrile generalized tonic-clonic seizures [14].

Despite the increased attention to *SCN1A*-related epilepsies, the diagnostic journey remains challenging and requires additional novel strategies and clinical advances focused on combining clinical examination with molecular diagnosis. As with any complex, enigmatic disease, diagnosing a patient with *SCN1A* variation requires a combined approach of clinical history, MRI, clinical EEG, and genotyping. While the clinical presentation may vary, the diagnostic testing provides us with the identification of *SCN1A* mutation [15]. Unfortunately, the treatment of *SCN1A*-related epilepsies is unsettling and includes aggressive seizure management while the developmental regression is persistent. Antiepileptic drugs (AEDs) are the primary management as they suppress the uncontrolled neuronal discharge due to the mutation in the *SCN1A* gene. Starting from sodium valproate and carbamazepine to stiripentol and cannabidiol, the options of AEDs have increased, offering a ray of hope for those who are suffering from intractable epilepsy [16].

The case of an 18-month-old child with a medical history of seizures and developmental delay illustrates the complexity of treating epilepsy and the significance of genetic testing. The patient initially presented with focal convulsion and later GTCS, which led to the use of AEDs for the management of seizures. Genetic studies are essential in developmentally normal, 2-15-month-old children having fever or vaccination-related unknown etiology for focal or generalized status epilepticus. Long-term therapy and monitoring help improve results among patients with *SCN1A* mutation [17,18].

## Conclusions

The identification of heterozygous mutation in the *SCN1A* gene in our patient provided us with valuable insights into the underlying cause of the intractable epilepsy and delay in the development of the patient. This case report highlights the importance of genetic testing in patients with intractable epilepsy to determine the molecular pathology of the disorder. The clinical course of the patient, with the need for multiple AEDs and finally giving some response to phenytoin, valproate, perampanel, and clobazam, underlines the difficulties in managing SCN1A-related seizures and the importance of a personalized treatment regimen for its resolution.
